# False reduction of an inguinal hernia treated by Kugel patch repair via an anterior approach

**DOI:** 10.1186/1471-2482-15-9

**Published:** 2015-02-02

**Authors:** Naoya Yamada, Atsushi Akai, Akihiko Seo, Yukihiro Nomura, Nobutaka Tanaka

**Affiliations:** Department of Surgery, Asahi General Hospital, 1326 I, Asahi-shi, Chiba, 289-2511 Japan

**Keywords:** Incarcerated inguinal hernia, Reduction en masse, CT imaging, Kugel patch repair

## Abstract

**Background:**

Reduction en masse of inguinal hernia is a rare condition following manual reduction of an unrecognized incarcerated inguinal hernia. The preoperative diagnosis and surgical treatment via an inguinal approach has been considered difficult.

**Case presentation:**

A 59-year-old man with lower abdominal pain was presented to our hospital. He was diagnosed reduction en masse of an inguinal hernia based on his CT findings which showed the presence a pre-peritoneal hernia sac containing the small bowel. An emergency operation via an anterior approach was performed and we found a hernial sac containing an incarcerated small bowel at the cranial and internal sides of the internal inguinal ring. Opening of the hernial sac revealed necrosis of the incarcerated small bowel and bowel resection was performed. Kugel patch was inserted into the pre-peritoneal space and the patient made an uneventful recovery.

**Conclusion:**

When it is accurately diagnosed, reduction en masse of an inguinal hernia can be treated with direct Kugel repair via an anterior approach.

## Background

False reduction of an inguinal hernia, also known as “reduction en masse”, is an extremely rare condition of an incarcerated inguinal hernia in which a hernia sac reduces into the preperitoneal space with its contents, but the retained bowel remains incarcerated and the obstruction persists [1–3]. Although the condition requires urgent surgical treatment, an accurate preoperative diagnosis has been difficult and a delay in diagnosis sometimes occurs because the inguinal hernia appears to be reduced [4–8].

Surgical treatment for reduction en masse of an inguinal hernia consists of relief of strangulation and hernia repair. In many reported cases of reduction en masse, exploratory laparotomy or laparoscopy was used because patients were preoperatively diagnosed with strangulated ileus of unknown origin. In those cases, reduction en masse was diagnosed on the basis of intraoperative findings.

However, recognition of cases and recent utilization of computed tomography (CT) imaging have made progress in accurate preoperative diagnosis of reduction en masse [1, 7, 8]. Accordingly, we herein report our experience of reduction en masse of inguinal hernia which was treated by Kugel patch repair via an anterior approach.

## Case presentation

A 59-year-old man presented to our hospital with lower abdominal pain and vomiting.

**Figure 1:**
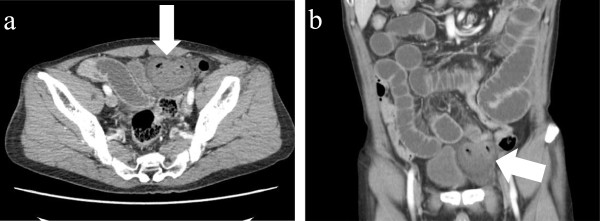
**CT findings scan of the patient.** Preoperative computed tomography findings of case showing small bowel obstruction with a transition point in the left inguinal groin. A segment of intestine is entrapped in a hernial sac protruding into the pre-peritoneal space; **a** *horizonal view*, **b** *frontal view*.

During a detailed interview, he said that he had noted a lump over his left groin for approximately 3 years that had initially been reducible by manipulation but had become progressively more difficult to reduce. Abdominal CT scan showed small bowel obstruction with a transition point in the left inguinal region (Figure 1). A segment of the intestine was entrapped within a hernial sac that was protruding into the pre-peritoneal space between the parietal peritoneum and anterior abdominal wall. The patient was diagnosed with a reduction en masse of an inguinal hernia and secondary mechanical small bowel obstruction.

**Figure 2:**
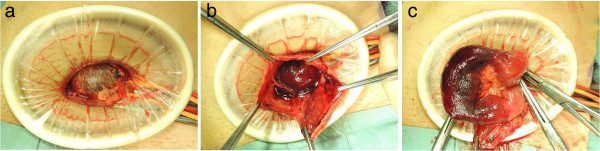
**Operational findings and surgical procedures.** The hernial sac containing the incarcerated small bowel is seen in the pre-peritonealspace at the cranial and internal sides of the internal inguinal ring **(a)**. Opening of the hernial sac revealed severe congestion and necrosis of the incarcerated small bowel **(b)**. The hernia neck is cut deeply to resect a 10-cm long portion of the necrotic incarcerated small bowel **(c)**.

An emergency operation was performed under general anesthesia. A 5-cm incision was made in the inguinal groin. The inguinal canal was opened, and the transverse fascia found to be weak. The pre-peritoneal space was spread widely, and an Alexis wound retractor (Applied Medical, Rancho Santa Margarita, CA, USA) was attached. We found a hernial sac containing an incarcerated small bowel at the cranial and internal sides of the internal inguinal ring (Figure 2a). These intraoperative findings confirmed proved the diagnosis of false reduction en masse of an inguinal hernia. Opening of the hernial sac revealed severe congestion and necrosis of the incarcerated small bowel (Figure 2b). The incarcerated small bowel was strangulated at the thickend hernia neck. Therefore, the hernia neck was cut deeply, and the small bowel was pulled out (Figure 2c). A 10-cm long portion of the small bowel was resected, along with the excess hernial sac, and the sac was closed with sutures. During these procedures, the pre-peritoneal space was spread wide enough to accommodate a direct Kugel patch. After the wound was extensively washed, an oval, 8 × 12 cm Bard Composix Kugel patch (Davol, Cranston, RI, USA) was inserted into the pre-peritoneal space and fixed to the internal oblique muscle. The patient made an uneventful recovery and was discharged on the fourth post-operative day. Six months have since passed with no sign of recurrence.

## Discussion

Reduction en masse of inguinal hernia is a rare condition and accurate preoperative diagnosis has been difficult. In previous reported cases of reduction en masse, exploratory laparotomy or laparoscopy was used because patients were preoperatively diagnosed with strangulated ileus of unknown origin. In those cases, reduction en masse was diagnosed on the basis of intraoperative findings and relief of strangulation and hernia repair could thus be performed. In our patient, we could made an accurate preoperative diagnosis of reduction en masse due to the careful medical history and mainly on the CT finding which showed the presence a pre-peritoneal hernia sac containing the small bowel and resembling to the previously reported cases [1, 7, 8].

In addition to the difficulty in establishing an accurate preoperative diagnosis, surgical treatment for reduction en masse via an inguinal approach has been considered difficult and has rarely been reported to date [5, 8]. Although transabdominal preperitoneal hernioplasty has been reported to be useful for reduction en masse [5, 7, 8], we considered that if an accurate preoperative diagnosis can be made, Kugel patch repair via an anterior approach would be an optimal surgical treatment.

The open peritoneal repair of an inguinal hernia was first described in the late 1990s, and Kugel patch inguinal hernia repair is one of the established treatment now, since it is less invasive with a relatively low recurrence rate, satisfactory patient comfort, and various ideal characteristics [9–11]. Based on our experiences, the key to the successful treatment of reduction en masse via anterior approach consists of spreading the pre-peritoneal space widely to fully expose the hernial sac, and then cutting down the neck of the hernial sac deeply enough to pull out the small bowel. After these procedures are performed, the pre-peritoneal space is spread wide enough to accommodate insertion of the Kugel patch.

For inguinal hernia surgery, the choice of laparoscopic surgery or open surgery with an inguinal approach is made according to an institute’s protocols. Not all institutes can perform emergency laparoscopic surgery under general anesthesia, and some patients are not good candidate for laparoscopic surgery. In addition, despite the development of minimally invasive procedures, laparoscopic surgery for small bowel obstruction itself carries the risk of perforation or other complications [12]. On the other hand, Kugel patch repair is sometimes associated with mesh-related infections, especially when bowel resection is involved [13, 14]. However, in this case, we avoided infection by using a wound retractor and extensive washing. Thus, Kugel patch repair via an anterior approach for reduction en masse can be an excellent choice with satisfactory outcomes.

## Conclusion

In conclusion, reduction en masse is a rare disease that can be accurately diagnosed by the collection of a detailed history and use of CT imaging and treated with direct Kugel repair via an anterior approach.

## Consent

Written informed consent was obtained from the patient for publicatin of this Case report and any accompanying images. A copy of the written consent is available for review by the Editor of this journal.

## Abbreviation

CT: computed tomography.
